# Genome Sequencing of *Ralstonia solanacearum* FQY_4, Isolated from a Bacterial Wilt Nursery Used for Breeding Crop Resistance

**DOI:** 10.1128/genomeA.00125-13

**Published:** 2013-05-09

**Authors:** Yi Cao, Baoyu Tian, Yanxia Liu, Liuti Cai, Hancheng Wang, Ning Lu, Maosheng Wang, Shenghua Shang, Zhengyou Luo, Junxiong Shi

**Affiliations:** Guizhou Tobacco Research Institute, Guiyang, Chinaa;; College of Life Sciences, Fujian Normal University, Fuzhou, Chinab

## Abstract

*Ralstonia solanacearum* strain FQY_4 was isolated from a bacterial wilt nursery, which is used for breeding crops for *Ralstonia* resistance in China. Here, we report the complete genome sequence of FQY_4 and its comparison with other published *R. solanacearum* genomes, especially with the strains GMI1000 and Y45 in the same group.

## GENOME ANNOUNCEMENT

*Ralstonia solanacearum*, the causal agent of globally dispersed bacterial wilt disease, infects an unusually wide range of plant species, and the harm is particularly serious in economically important crops, such as tomatoes, tobacco, and potatoes. (1). Resistance to bacterial wilt is an important characteristic to evaluate in the well-characterized cultivars in these crop-breeding programs. *R. solanacearum* strain FQY_4 was isolated from a bacterial wilt nursery, which was built for breeding crop resistance in 1997 in the Guizhou province in southwest China. Here, the complete genome of the pathogen-testing index strain FQY_4 has been sequenced, annotated, and compared to the genomes of other sequenced *R. solanacearum* strains (2, 3).

The nucleotide sequence of the FQY_4 genome was determined from a paired-end library with an average insert size of 500 bp and a mate-pair library with average insert size of 2,000 bp, using the Illumina HiSeq 2000. The trimmed reads were *de novo* assembled into 286 contigs (>200 bp) by using Velvet 1.2.01 with Kmer 55 (4), providing 51-fold coverage. Contigs were then divided into chromosome and megaplasmid parts by using *R. solanacearum* strain GMI1000 as the reference genome. The relationship among contigs was determined according to the mate-pair information from the mapping protocol (5–7) and finally was organized in linear draft genome sequences. Putative protein-coding sequences were predicted by Glimmer (8). Functional annotation was based on the BLASTp results against the GenBank NR and Pfam databases. Phylogenetic and comparative genomic analyses were carried out using MEGA4 based on *egl* and *hrpB* sequences, in Mauve and the RAST server (9–11).

In total, a complete chromosome sequence of 3,715,422 bp and a draft megaplasmid sequence (with one gap) of 2,089,828 bp were generated, resulting in the 5.8-Mb FQY_4 genome. The average G+C content of the FQY_4 genome is 66.82%. The entire genome contains 5,153 coding sequences, 62 tRNA genes, and 9 complete rRNA loci. Compared against the 10 previously published *R. solanacearum* genomes, strains *R. solanacearum* GMI100 and Y45 were most closely related to FQY_4, which is consistent with the phylogenetic analysis results that assign these three strains to the same phylotype I group. Of the 5,153 FQY_4 coding sequences, 4,278 and 4,204 coding sequences have 97 to 100% identity to strains GMI100 and Y45, respectively. There are 526 FQY_4-specific genes in comparison to strain GMI100 and 356 specific genes compared to strain Y45. Interestingly, a phage-sourced death on curing protein (Doc toxin), which is part of a two-protein operon with prevents-host-death (Phd) that forms a bacterial toxin-antitoxin system, is present in FQY_4 but absent in both strains GMI100 and Y45 (12); also, a type IV secretory pathway VirD4 component-like protein is absent from FQY_4. Whole-genome alignments revealed a large variability in the organization of the genomes of three phylotype I strains, including many rearrangements and inversions. Genome sequencing of FQY_4 provided us an example to explore how strains with geographical and pathogenic variation emerged and evolved and how they acquired new traits, such as resistance or pathogenicity, and pathogenic variation in different host cultivars.

### Nucleotide sequence accession numbers.

*R. solanacearum* strain FQY_4 chromosome and megaplasmid sequences were deposited in GenBank under accession no. CP004012 and CP004013, respectively.
